# Open- vs. closed-chest pig models of donation after circulatory death

**DOI:** 10.3389/fcvm.2024.1325160

**Published:** 2024-06-13

**Authors:** Valentin Hubacher, Manuel Egle, Selianne Graf, Maria Arnold, Adrian Segiser, Maria Nieves Sanz, Daniela Casoni, Luisana Garcia Casalta, Kay Nettelbeck, Maks Mihalj, Matthias Siepe, Alexander Kadner, Sarah Longnus

**Affiliations:** ^1^ Department of Cardiac Surgery, Inselspital, Bern University Hospital, University of Bern, Bern, Switzerland; ^2^ Department for BioMedical Research, University of Bern, Bern, Switzerland; ^3^ Graduate School for Cellular and Biomedical Sciences, University of Bern, Bern, Switzerland; ^4^ Experimental Surgery Facility (ESF), Experimental Animal Center, Faculty of Medicine, University of Bern, Bern, Switzerland; ^5^ Department of Advanced Cardiopulmonary Therapies and Transplantation, University of Texas Health Science Center at Houston, Texas Medical Center, Houston, TX, United States

**Keywords:** donation after circulatory death (DCD), DCD heart transplantation, DCD pig model, open- vs. closed-chest physiology, warm ischemia

## Abstract

**Background:**

During donation after circulatory death (DCD), cardiac grafts are exposed to potentially damaging conditions that can impact their quality and post-transplantation outcomes. In a clinical DCD setting, patients have closed chests in most cases, while many experimental models have used open-chest conditions. We therefore aimed to investigate and characterize differences in open- vs. closed-chest porcine models.

**Methods:**

Withdrawal of life-sustaining therapy (WLST) was simulated in anesthetized juvenile male pigs by stopping mechanical ventilation following the administration of a neuromuscular block. Functional warm ischemic time (fWIT) was defined to start when systolic arterial pressure was <50 mmHg. Hemodynamic changes and blood chemistry were analyzed. Two experimental groups were compared: (i) an open-chest group with sternotomy prior to WLST and (ii) a closed-chest group with sternotomy after fWIT.

**Results:**

Hemodynamic changes during the progression from WLST to fWIT were initiated by a rapid decline in blood oxygen saturation and a subsequent cardiovascular hyperdynamic (HD) period characterized by temporary elevations in heart rates and arterial pressures in both groups. Subsequently, heart rate and systolic arterial pressure decreased until fWIT was reached. Pigs in the open-chest group displayed a more rapid transition to the HD phase after WLST, with peak heart rate and peak rate-pressure product occurring significantly earlier. Furthermore, the HD phase duration tended to be shorter and less intense (lower peak rate-pressure product) in the open-chest group than in the closed-chest group.

**Discussion:**

Progression from WLST to fWIT was more rapid, and the hemodynamic changes tended to be less pronounced in the open-chest group than in the closed-chest group. Our findings support clear differences between open- and closed-chest models of DCD. Therefore, recommendations for clinical DCD protocols based on findings in open-chest models must be interpreted with care.

## Introduction

Despite the progress in the treatment of advanced heart failure with mechanical circulatory support systems and medical management, orthotopic allogeneic heart transplantation remains the gold standard. Due to the shortage of donor organs, the waitlist time and mortality remain high (1). To mitigate the mismatch between donor organ supply and demand, new ways have been sought to expand the donor pool via heart transplantation with organ donation after circulatory determination of death (DCD). Some reports have observed a considerable increase in annual transplant numbers by 15%–48% by expanding the donor pool by including DCD organs (2, 3). This is of particular clinical importance, as a growing body of evidence supports the feasibility and non-inferiority of DCD heart transplantation, with comparable graft function and clinical outcomes after heart transplantation between DCD and conventional donation after brain death (DBD) (2–5).

Given the excellent results with DCD heart transplantation to date, it now makes sense to turn our attention to optimizing clinical protocols. Indeed, the reintroduction of DCD heart transplantation involves varying clinical protocols with a lack of agreement over key definitions. While warm ischemic time generally represents the time from withdrawal of life-sustaining therapy (WLST) to reperfusion, no consensus exists on the definition of which objective circulatory parameters should be fulfilled for the functional warm ischemic time (fWIT) (6, 7). Since these definitions have a major impact on the assessment of organ viability, clarification of their defining thresholds is needed. Similarly, various preclinical DCD models have been reported (8), and it is important to fully characterize these models and carefully interpret the corresponding data to most effectively translate promising preclinical findings into clinical practice.

The concept of DCD heart transplantation brings enormous opportunity but is also associated with a broad set of challenges. The inevitable warm ischemia time to which the potential donor organs are exposed during the course of circulatory arrest is of particular concern (9). Previous models have shown stereotypical changes in physiology after WLST, which occur in three phases. The first phase marks a decline in blood oxygenation and pressure, heart rate (HR), and cardiac output, with an increase in lactate and potassium (K) (10). Before complete cessation of the circulation, the blood pressure increases again during the hyperdynamic (HD) phase. During this second phase, a 120-fold increase in adrenaline and a 400-fold increase in noradrenaline have been reported (11). The catecholamine surge occurs in parallel with an increased heart rate, cardiac output, systolic pressure, and the release of free fatty acids (FFAs) into the circulation (12). In the final phase, a decrease in blood pressure and heart rate ensues until circulatory arrest is declared. Strategies to limit cellular stress caused by warm ischemia require an understanding of the pathophysiological processes that follow WLST. Information about this post-withdrawal phase in human DCD donors is limited as antemortem interventions are prohibited in many countries, such as performing invasive measurements for monitoring. As a result, translational animal models are of central importance for improving protocols in clinical DCD.

The aim of this translational study was to identify the effects of protocol variations in experimental porcine models of DCD by comparing open- and closed-chest models. We aimed to focus on the perimortem pathophysiology to extend our knowledge of changes occurring during this period to help guide the optimization of clinical protocols for DCD heart transplantation in clinical practice and to aid in the interpretation and integration of findings obtained from various preclinical models of DCD.

## Materials and methods

### Animals

Male Edelschwein pigs with a mean body weight of 56.4 kg were used in this study. All experimental procedures were performed in compliance with the European Convention for Animal Care and approved by the Swiss animal welfare authorities and the Ethics Committee for Animal Experimentation, Berne, Switzerland (approval number BE68/2019).

This study was performed in the context of a larger series of animals that served in the investigation of ischemic tolerance and graft biomarkers using predefined fWIT durations that will be published separately. To investigate differences between open- and closed-chest porcine models and to provide clarity for reproducibility, we generated a separate manuscript to fully characterize this model, which should also help in the interpretation of data and relevance in similar models.

### Experimental design

**Figure 1:**
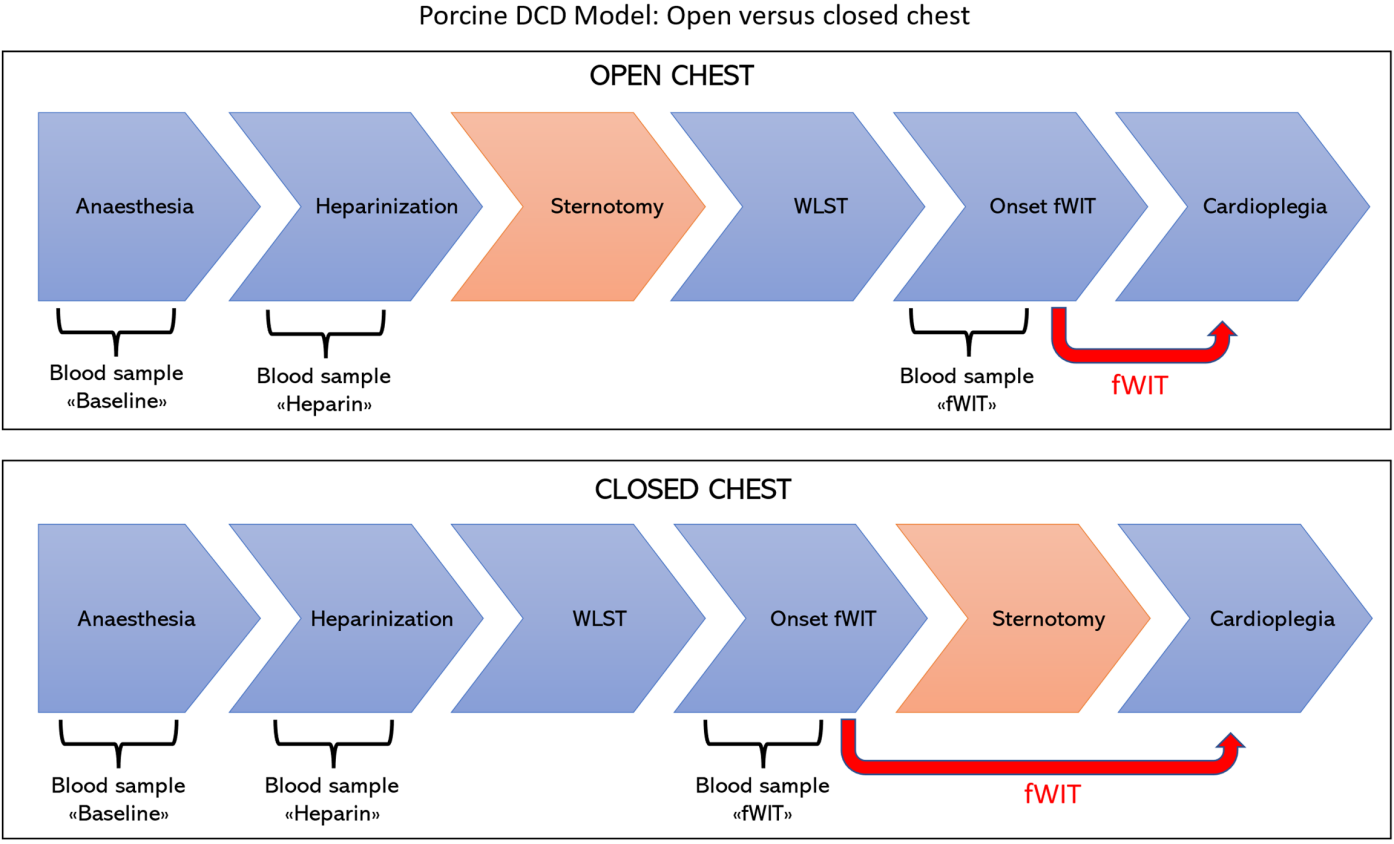
Experimental design and model of DCD. In the open-chest group, sternotomy was performed prior to withdrawal of life-sustaining therapy (upper panel) in line with protocols frequently used in preclinical research models. In the closed-chest group, sternotomy was performed after withdrawal of life-sustaining therapy (lower panel) as generally occurs in clinical practice during DCD heart transplantation.

The experimental design and surgical protocols for the DCD model are presented in Figure 1. Animals were randomly allocated to one of the two groups according to whether sternotomy was performed before or after WLST and designated as the open-chest or closed-chest group, respectively.

In the open-chest group, sternotomy was performed before WLST to allow rapid application of cold cardioplegia and removal of the heart directly at the onset of fWIT. Pigs in the closed-chest group were subjected to a predefined duration of functional warm ischemia (20–30 min) and thus did not require prior sternotomy for rapid procurement.

### Anesthesia and surgical monitoring

Pigs were sedated with ketamine (15 mg/kg), midazolam (0.5 mg/kg), and methadone (0.2 mg/kg) administered intramuscularly behind the ear. Additional midazolam (up to a maximum of 1 mg/kg) was administered if sedation was deemed clinically insufficient after at least 15 min from the first injection. An intravenous cannula was placed in a marginal ear vein, and a Ringer lactate infusion started at 5 ml/kg/h. Induction of general anesthesia was achieved with propofol to effect (1–2 mg/kg) and ketamine (1 mg/kg). After endotracheal intubation, anesthesia was deepened and maintained with total intravenous anesthesia (TIVA) based on propofol (12–18 mg/kg/h) and fentanyl (5 μg/kg/h). Additional boluses of fentanyl (5–10 μg/kg) or ketamine (0.5–1 mg/kg) and/or increases in the infusion rate were implemented as required. Spontaneous intermittent mandatory ventilation was started after tracheal intubation in a volume-controlled, pressure-limited mode using a positive end-expiratory pressure (PEEP) of 5 cmH_2_O, an FiO_2_ of 0.50, and a tidal volume of 10 ml/kg body weight, while the respiratory rate was adjusted targeting a PaCO_2_ of 40–45 mmHg. In addition to intravenous analgesia, 0.75% epidural or spinal ropivacaine (1.5 mg/kg) and morphine (0.1 mg/kg) were administered prior to the operative procedure at the lumbosacral space to optimize analgesia, diminish autonomic response, and minimize requirements for intravenous anti-nociception. Rescue analgesia (fentanyl 5 μg/kg) was administered for any increase of at least 20% of the mean arterial pressure (MAP) compared to the baseline values, recorded before the first incision in the cervical ventral region. Propofol, fentanyl, and Ringer lactate infusions were continued until the end of heart procurement.

During general anesthesia, continuous monitoring of heart rate, heart rhythm (ECG), respiratory rate, oxygen arterial saturation, capnography, invasive blood pressure (from a peripheral artery and after its cannulation from the carotid artery), esophageal temperature, inspired and expired fraction of gases (air, etCO_2_), and central venous pressure (CVP) were provided through a multimodular monitor (S/5 Critical Care Monitor®; Datex-Ohmeda, GE Healthcare, Helsinki, Finland). Brain activity was monitored by an indexed EEG monitor (Sedline Sedation Monitor, Masimo, USA). A blood gas analysis was carried out before commencing the DCD protocol (see below). An MAP of 65 mmHg was targeted before discontinuing mechanical ventilation, and Ringer's lactate boluses were administered as necessary. General anesthesia was carried out under the supervision of a boarded veterinary anesthesiologist.

### DCD protocol

**Figure 2:**
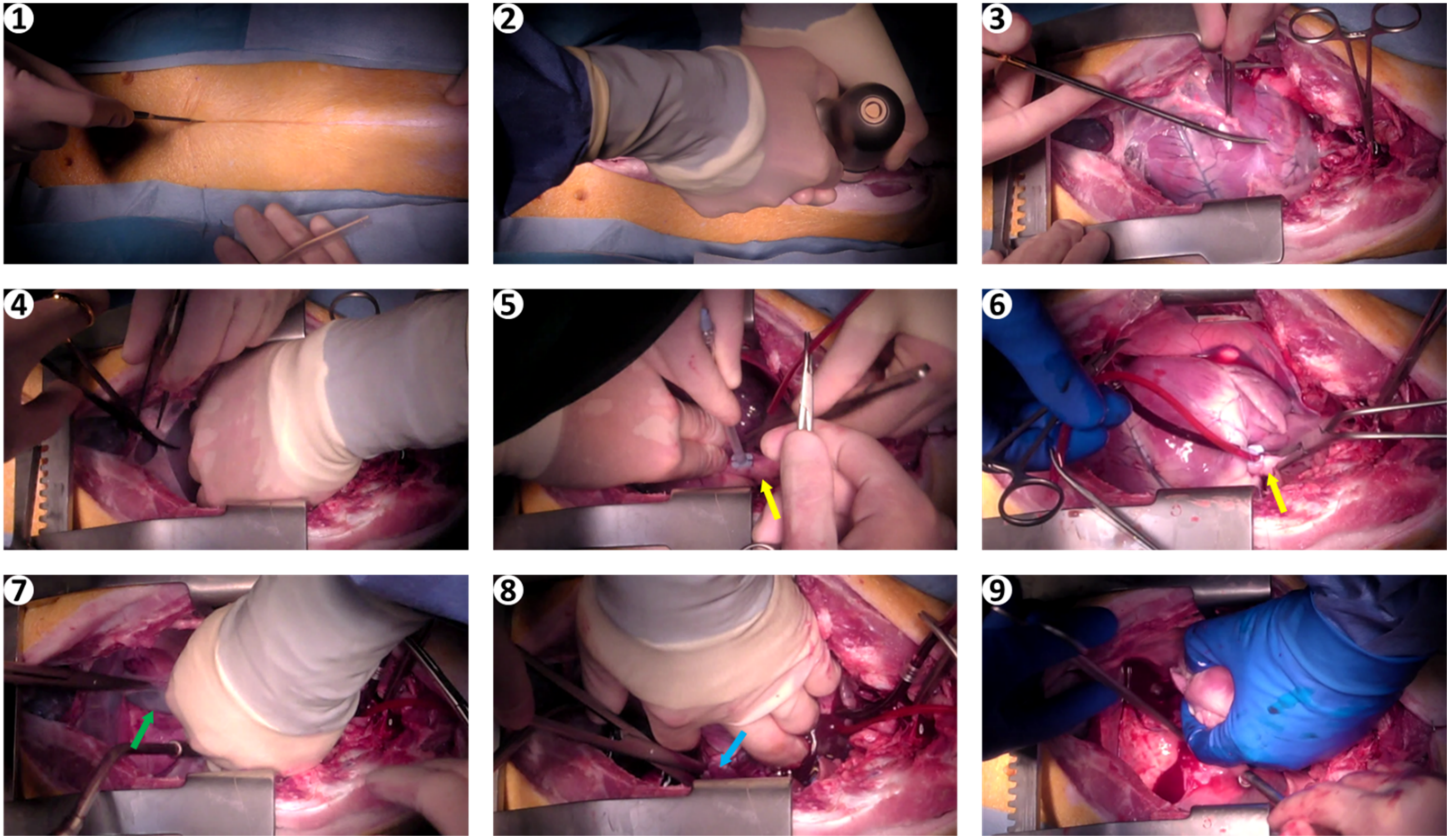
Images of surgical procedure. (1) skin incision; (2) sternotomy; (3,4) opening of the pericardium; (5) placement of the cardioplegia needle in the aortic root; (6) cross clamping of the ascending aorta; (7) incision of the inferior vena cava; (8) incision of the left atrial appendage; (9) heart removal. Yellow arrows, aorta; green arrow, inferior vena cava; blue arrow, left atrial appendage.

The common carotid artery and external jugular vein on the right side were cannulated, amiodarone (5 mg/kg) and unfractionated sodium heparin (10,000 IU/animal) were administered IV before commencing the DCD protocol. To simulate WLST, mechanical ventilation was discontinued after the administration of atracurium (1 mg/kg bolus, followed by a continuous infusion rate of 0.2 mg/kg/h). The endotracheal tube remained connected to the breathing system and unclamped. Ventilation was always stopped at the end of a respiratory cycle. The onset of fWIT was defined as the time when systolic arterial pressure (SAP) dropped below 50 mmHg. Pathophysiological monitoring and biochemical measurements in blood were performed up until fWIT onset in the two experimental groups with a differing sequence of surgical interventions, as shown in Figure 2 and described below.

An electric monopolar radiofrequency coagulation device was used to prevent blood loss in the skin and subcutaneous tissue. After sternotomy, bleeding from the periosteum was stopped with the same device. If needed, an Ostene bone hemostat (Baxter Healthcare Corporation, Westlake Village, USA) was used to stop more severe bleeding from the sternal spongiosa, but this was rarely necessary. The rest of the procedure, namely, transection of the pericardium and removal of the thymus, was also performed with the monopolar cautery knife mentioned above and required no further hemostatic measures. Any small injuries in the area of the great vessels could be repaired locally with monofilament sutures; no major injuries occurred. Overall, there was no unforeseen intraoperative blood loss in any of the pigs prior to fWIT. In the open-chest group, in which sternotomy and placement of the cardioplegic needle (CPN) in the ascending aorta were performed before WLST, hearts were procured as rapidly as possible after fWIT onset.

In the closed-chest group, in which sternotomy and placement of the cardioplegic needle in the ascending aorta were performed after WLST and fWIT onset, the time of circulatory arrest, defined as pulse pressure <5 mmHg, was noted.

Data reported in this manuscript are limited to the pre- and post-WLST phases until and including the onset of fWIT.

### Hemodynamic measurements

Arterial blood pressure was invasively monitored by catheterizing the right common carotid artery. Central venous pressure was determined using a catheter placed in the right external jugular vein. For the evaluation of peripheral oxygen saturation (SpO_2_), a pulse oximeter was attached to the pig's lower lip. Data were recorded in 5-s intervals throughout the procedure. At WLST, a handheld timer was started to record time to fWIT. The measured time interval was used to reconstruct the timing of WLST in the recorded anesthesia data, which is the basis of time calculations in this report. The rate-pressure product (RPP) was calculated as the product of heart rate and SAP. RPP was used to evaluate the extent of sympathetic circulatory response to hypoxia after WLST, referred to as the HD phase), which was defined to start with five consecutive RPP measurements above the mean pre-WLST value and end with five consecutive measurements below this pre-WLST mean. Pre-WLST mean values were calculated as the average of all measurements during the 60 s prior to WLST initiation, except for one heart in which representative values were taken 10 s prior to WLST.

### Blood gas and biochemistry analysis

Three 10 ml arterial blood samples were obtained: (i) baseline (approximately 15 min before heparin administration); (ii) heparin (1 min after heparin administration); (iii) fWIT (immediately upon fWIT onset). Blood chemistry analysis was performed immediately, and the remaining volume was used to prepare plasma, which was frozen at −80°C for later analysis.

Blood gas analysis was carried out using a Cobas® b 123 point-of-care analyzer (Roche, Basel, Switzerland). The FFA content in the blood plasma was determined using the Free Fatty Acid Quantitation Kit (Sigma-Aldrich, Missouri, USA) according to the manufacturer's instructions. Noradrenaline and adrenaline concentrations in blood plasma were also quantified using the Epinephrine/Norepinephrine ELISA kit (Abnova, Taipeh City, Taiwan) according to the manufacturer's instructions. Troponin I (Life Diagnostics Inc., West Chester, USA) and heart-type fatty acid-binding protein (Life Diagnostics Inc., West Chester, USA) in plasma samples were measured according to the manufacturers’ instructions.

### Data analysis

All data were analyzed using GraphPad Prism (version 7.0, GraphPad Software Inc., La Jolla, CA, USA). Unless otherwise stated, values are reported as mean ± standard deviation.

The Kruskal–Wallis test was performed for an overview of differences between experimental groups, and when significant overall results were observed, comparisons between groups of interest were performed with Mann–Whitney tests. The Spearman rank correlation test was used to investigate correlations among variables. Two-tailed *p*-values were adjusted for multiple comparisons (modified, sequential, rejective Bonferroni procedure) (13). Corrected *p*-values were considered statistically significant if <0.05.

## Results

Eighteen pigs were included in this study, comprising two experimental groups: (i) open chest at WLST (*n* = 7) and (ii) closed chest at WLST (*n* = 11). No difference in baseline characteristics between experimental groups was observed (Table 1). Biochemical measurements (lactate, hemoglobin, glucose) were performed using the baseline sample.

**Table 1:** Baseline characteristics of experimental groups.

	Open chest	Closed chest
Sample size (number of animals)	7	11
Age (months)	3.9 ± 0.2	3.9 ± 0.5
Body weight (kg)	56.1 ± 6.3	56.6 ± 7.4
Blood lactate (mmol/L)	1.4 ± 0.4	1.4 ± 0.3
Total hemoglobin (g/dl)	8.9 ± 1.0	9.2 ± 0.6
Blood glucose (mmol/L)	5.3 ± 0.5	5.2 ± 0.6

Biochemical measurements (lactate, hemoglobin, glucose) were performed using the baseline sample.

**Figure 3:**
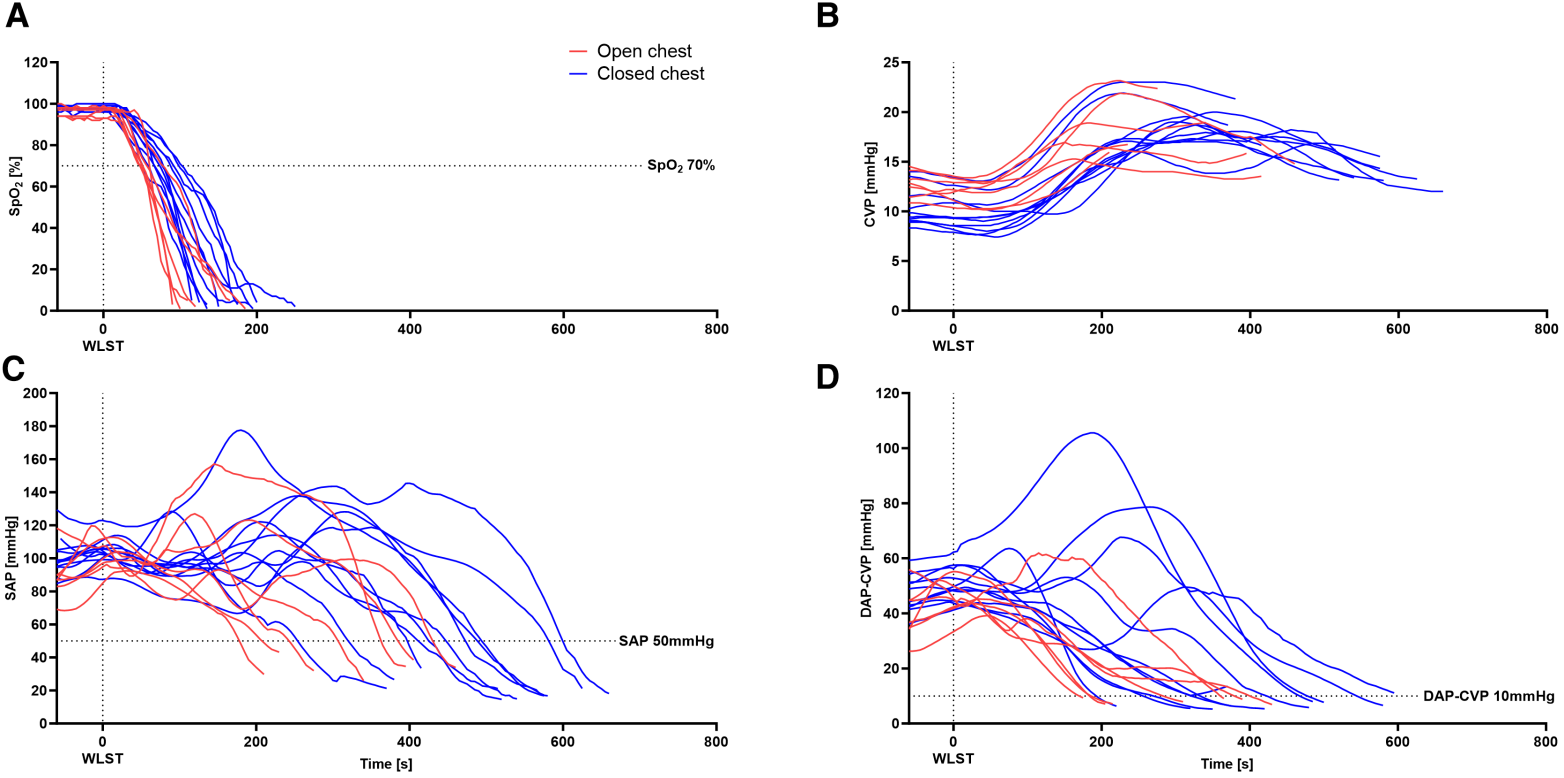
Hemodynamic changes in the porcine model of DCD. Progression of SpO_2_ (**A**), CVP (**B**), SAP (**C**), and the difference between DAP and CVP (**D**) from WLST to the onset of functional warm ischemic (defined as systolic arterial pressure ≤50 mmHg). *n* = 7–11.

Hemodynamic changes during the progression from WLST to fWIT are presented in Figure 3. WLST was followed by a rapid decline in SpO_2_ (Figure 3A) and a subsequent cardiac hyperdynamic period with temporary elevations in the heart rate and arterial pressure (Figure 3B) in both groups. Subsequently, the heart rate and systolic and diastolic pressures decreased, whereas the CVP increased gradually in both groups (Figure 3C), contributing to a diminishing pressure difference between diastolic arterial pressure (DAP) and CVP (Figure 3D). In general, progression to fWIT occurred more rapidly in the open-chest model than in the closed-chest model.

**Figure 4:**
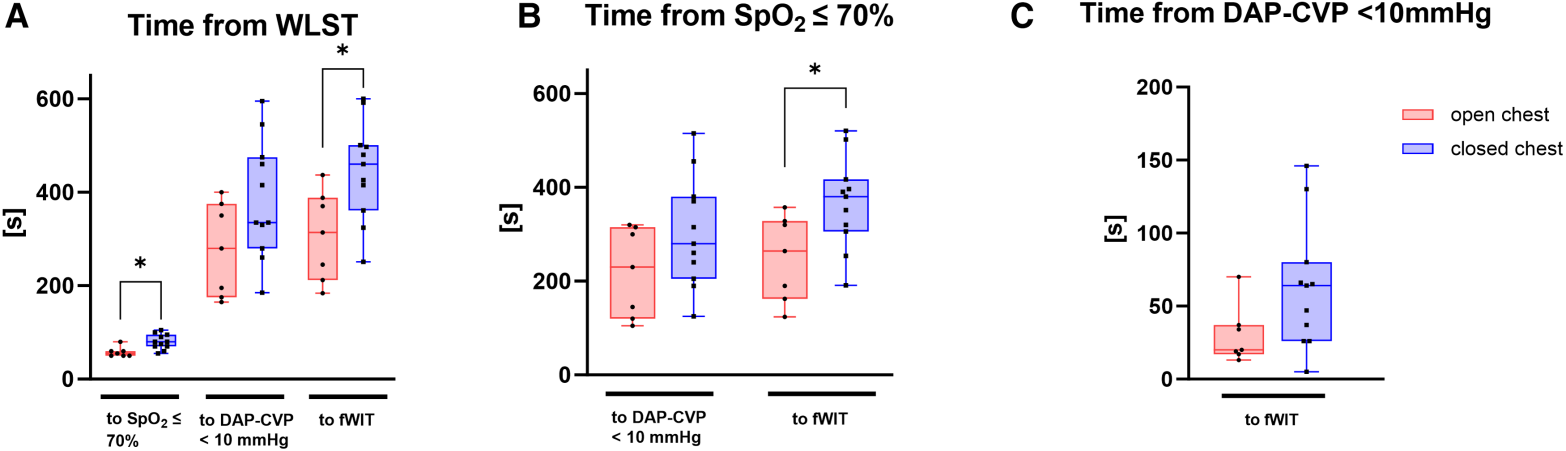
Time intervals of interest starting from (**A**) WLST, (**B**) SpO_2_ ≤ 70% and (**C**) DAP-CVP < 10 mmHg. Data are presented as medians, 25–75 percentiles, and ranges. DAP–CVP, difference between DAP and CVP; fWIT, start of functional warm ischemic time defined as systolic arterial pressure ≤50 mmHg. *p < 0.05; *n* = 7–11.

Pigs in the open-chest group demonstrated a more rapid decline in vital parameters. Median times from WLST to SpO_2_ < 70% or to fWIT were significantly lower in the open-chest pigs than in closed-chest pigs (*p *< 0.05 for both; Figure 4A). The mean duration from WLST to the onset of fWIT was 5.3 ± 1.5 min in the open-chest group vs. 7.4 ± 1.9 min in the closed-chest group. The interval between the time point at which SpO_2_ reached 70% and either the time point when the difference between DAP and CVP fell below 10 mmHg (Δ10 mmHg) or the fWIT onset was also shorter in open-chest pigs than in closed-chest pigs, reaching statistical significance for only fWIT onset (*p *< 0.05; Figure 4B). Although there was also a tendency for a shorter duration from Δ10 mmHg to fWIT in the open-chest group compared to the closed-chest group, this did not reach statistical significance (Figure 4C).

**Figure 5:**
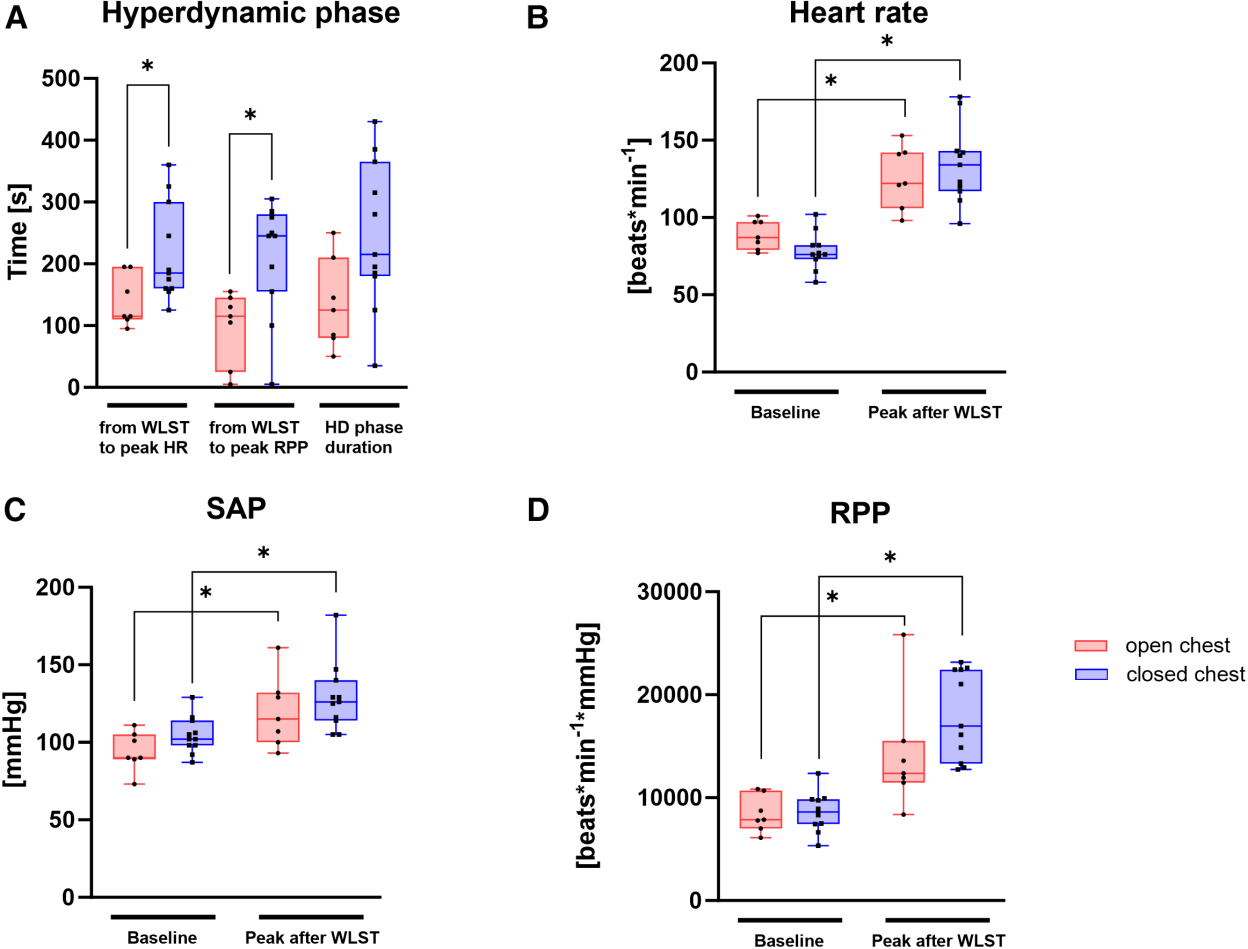
Hyperdynamic phase in the open- and closed-chest experimental groups. (**A**) Progression from WLST to peak HR and RPP, as well as HD duration; values at baseline and peak HD for heart rate (**B**), SAP (**C**), and RPP (**D**). RPP, SAP × heart rate. Data are presented as medians, 25–75 percentiles, and ranges; *p < 0.05; *n* = 7–11.

Pigs in the open-chest group displayed a more rapid transition to the HD phase after WLST, with peak HR and RPP occurring significantly earlier (Figure 5A). Although the HD phase duration tended to be shorter (Figure 5A) and less intense (the tendency for a lower peak RPP; Figure 5B) in the open-chest group than in the closed-chest group, these differences were not statistically significant. HR, SAP, and RPP were significantly increased after WLST compared to the baseline measurement in both groups (Figures 5B–D).

**Figure 6:**
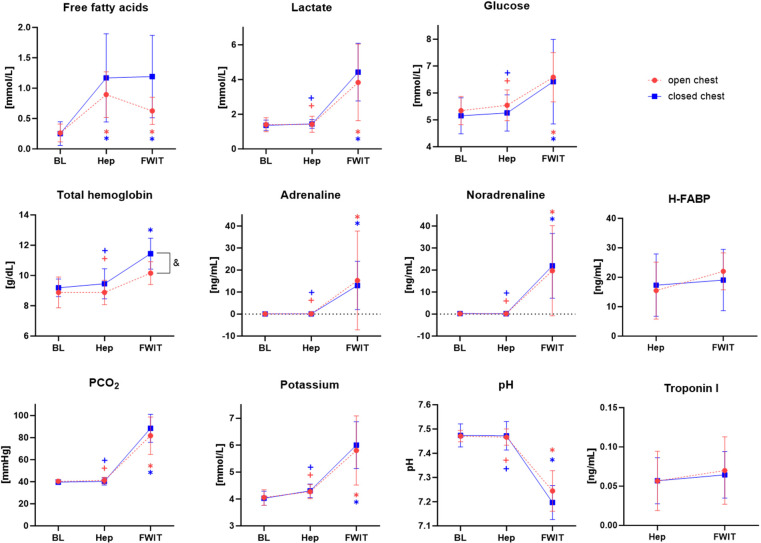
Mean blood values for the open- and closed-chest groups at baseline (BL), heparin (Hep), and fWIT onset (fWIT) time points. *p < 0.05 vs. corresponding BL value; ^+^*p* < 0.05 vs. corresponding fWIT value; ^&^*p* < 0.05 open- vs. closed-chest group; *n* = 7–11.

Figure 6 displays the blood analysis results from the three predefined blood sampling time points. Interestingly, FFA was significantly increased at heparin and fWIT time points compared to baseline in both experimental groups (*p* < 0.05); however, levels tended to be lower in open-chest pigs than in closed-chest pigs at fWIT. In addition, total hemoglobin (tHB) tended to increase at fWIT compared to baseline and heparin time points in both experimental groups but was significantly higher in the closed-chest group than in the open-chest group at fWIT onset (*p* < 0.05). Values for lactate, glucose, adrenaline, noradrenaline, CO_2_ partial pressure (pCO_2_), and potassium were significantly increased at fWIT compared to those at baseline and heparin time points and were similar between experimental groups. Correspondingly, blood pH was significantly decreased at fWIT compared to that at baseline and heparin time points, again showing similar trends between experimental groups. Markers of myocardial cell death, troponin I, and heart-type acid-binding protein (H-FABP) were similar at heparin and fWIT time points, and no differences were observed between experimental groups.

**Figure 7:**
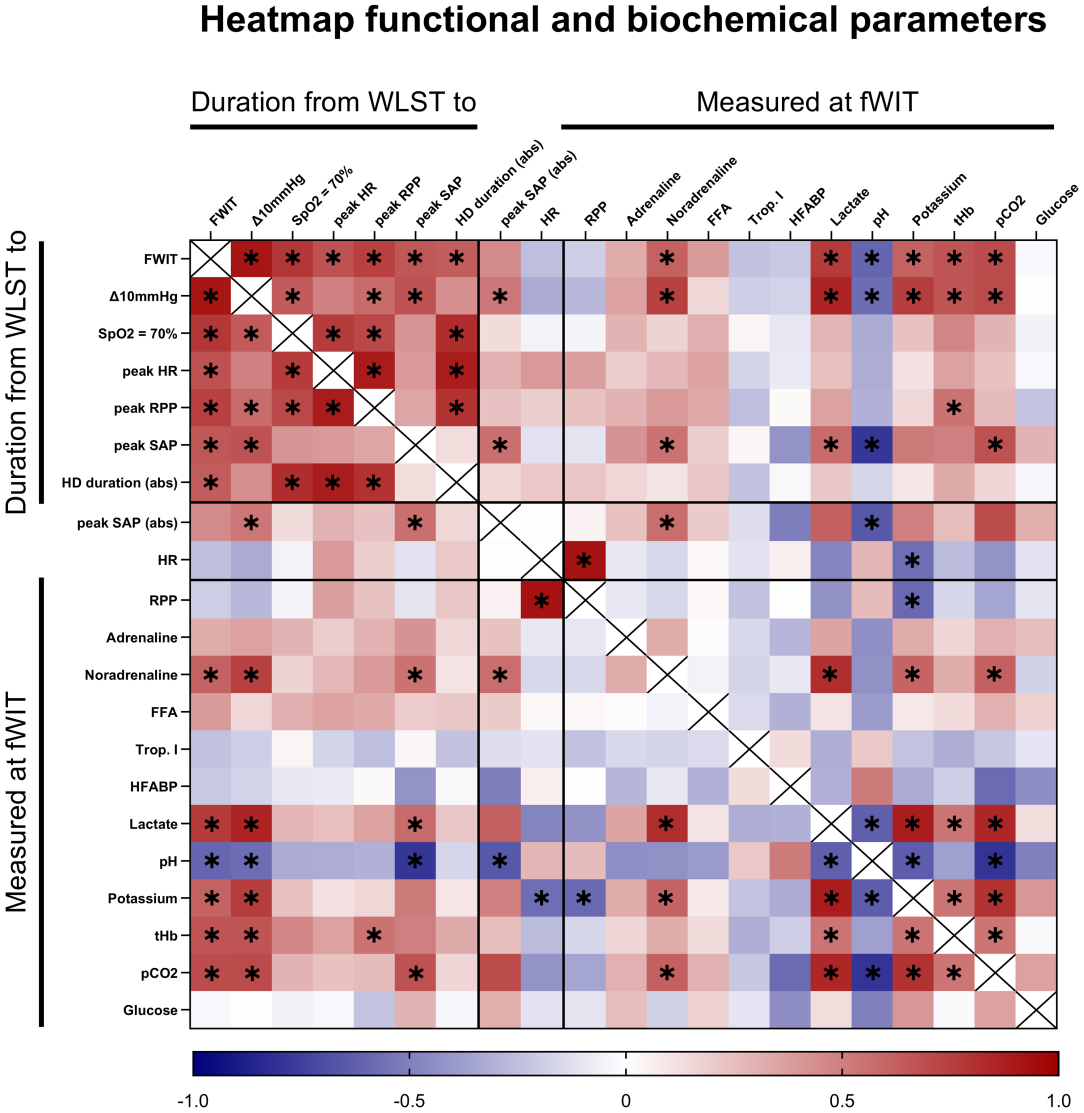
Heatmap of correlation analysis including all hearts (open and closed chest) between various time intervals starting from WLST, hyperdynamic values, and biochemical variables measured at fWIT onset. Δ10 mmHg, difference between diastolic arterial pressure and central venous pressure of 10 mmHg; RPP, rate-pressure product (SAP × HR); Trop. I, troponin I. abs, indicates absolute values. The color scale indicates the rho value. *p < 0.05; *n* = 18.

Correlations between key time points in the progression to fWIT onset, hemodynamic changes, and biochemical measurements were investigated for all hearts together (Figure 7). As expected, the times required to reach key physiologic progression landmarks (fWIT, 70% SpO_2_, Δ10 mmHg) were frequently positively intercorrelated. In line with these findings, the times required to reach these progression landmarks were also positively correlated with times from WLST to markers of the hyperdynamic phase (peak heart rate, peak SAP, and peak RPP). Interestingly, the times required to reach these progression landmarks were also positively correlated with indicators of hyperdynamic phase intensity (hyperdynamic phase duration and peak SAP). Among measurements in blood at fWIT onset, several other variables (e.g., noradrenaline, lactate, and pCO_2_) correlated positively and blood pH correlated negatively with key progression measurements (time from WLST to fWIT, Δ10 mmHg, and peak SAP). Interestingly, blood noradrenaline concentration and pH correlated positively and negatively, respectively, with peak SAP values. Furthermore, noradrenaline correlated positively with lactate, potassium, and pCO_2_. However, no statistically significant correlations were observed for blood adrenaline concentrations. Lactate correlated negatively with pH and positively with potassium, total hemoglobin, and pCO_2_. In addition, pH negatively correlated with potassium and pCO_2_, while potassium positively correlated with total hemoglobin and pCO_2_. No significant correlations were observed for blood concentrations of free fatty acids, glucose, troponin I, or H-FABP.

## Discussion

Understanding the pathophysiological changes surrounding heart donation with donation after circulatory death is essential for the identification and development of strategies to ensure optimal cardiac graft quality in both preclinical and clinical settings and ultimately to improve outcomes for patients undergoing heart transplantation with DCD. Information about this perimortem phase in human DCD donors is limited, as legal and ethical considerations limit invasive monitoring. In this study, we characterized heart donation with controlled DCD in a porcine model, focusing on the interval between WLST and the onset of fWIT. Two experimental groups were included, with and without sternotomy prior to WLST. Animals that underwent sternotomy prior to WLST demonstrated a more rapid progression in nearly all vital parameters, resulting in an earlier onset of fWIT compared to the group with a closed chest during the same interval. We expect a transient hyperdynamic phase following WLST with elevated heart rate and elevated or sustained blood pressure. In the open-chest group, this response to hypoxia occurs more rapidly than in the closed-chest group. Furthermore, the peak arterial pressure, and RPP and hyperdynamic phase duration tended to be lower in the open-chest group than in the closed-chest group. Of particular interest, we observed changes in several blood chemistry variables during the period between WLST and fWIT onset that correlate with key measurements of hemodynamic progression during this time. In general, despite not observing significant differences in markers of myocardial cell death (heart-type fatty acid-binding protein, troponin I), our findings support the concept that hearts in open-chest conditions are exposed to potentially less damaging conditions than in closed-chest conditions. This conclusion is drawn from the progression to fWIT occurring more rapidly, trends for a less intense hyperdynamic period, and potentially shorter exposure to altered blood chemistry in the open-chest group. Importantly, in a clinical DCD setting, patients will have closed chests in most cases, which means that findings in open-chest models must be interpreted in this context. Taken together, our findings support apparent differences in open- and closed-chest models of DCD, which are important as many of the recommendations for clinical DCD protocols are based on animal models with an open chest (7, 14).

Multiple factors could contribute to the differing dynamics in open- vs. closed-chest conditions following WLST in this study. For example, if a neuromuscular block was insufficient and some respiratory effort was still present, the efficiency of respiration would likely be higher in a closed chest. However, no lung ventilation was observed with our continuously monitored expiratory CO_2_. Further, the negative pressure in the thorax is eliminated with sternotomy. During positive pressure ventilation, this should have little influence on the expansion of the lung; but after the cessation of ventilation, the positive end-expiratory pressure provided by the ventilator no longer prevents the collapse of the lung. A diminished residual gas volume could accelerate progression after WLST. Finally, the pressure difference between the abdomen and the thoracic cavity is altered with sternotomy, which may lead to a diminished venous return to the right atrium. Our findings demonstrate that the main difference in progression appears to lie within the immediate post-withdrawal interval between WLST and the peak hemodynamic response. These findings are in agreement with more rapidly depleted oxygen availability in the open-chest group, as mentioned above.

A surge in catecholamine release following WLST and decreasing blood oxygen saturation has been reported in other preclinical DCD models (10–12, 15). We observed an increase in adrenaline and noradrenaline at fWIT onset. In absolute terms, catecholamine levels in our model were lower than those reported in the literature (11). This discrepancy may result from the use of spinal anesthesia prior to WLST in our study. The use of low spinal anesthesia with no suppression of efferent adrenergic tone has been demonstrated to prevent the neuroendocrine responses to surgical stress in human patients (16). As might be expected, given its effects on blood pressure, circulating noradrenaline concentrations at fWIT correlated positively with time from WLST to fWIT and other key progression indicators, as well as with the peak heart rate and peak SAP. These findings suggest that higher noradrenaline levels at fWIT led to a slower progression to fWIT and a more severe hyperdynamic phase, which would be expected to be more damaging for the heart. Interestingly, despite different progression rates to fWIT and hyperdynamic phase intensities between open- and closed-chest groups, blood noradrenaline concentrations were similar at fWIT (although we cannot exclude differences between groups at time points that were not measured). Taken together, these findings suggest that the slower decline in SpO_2_ in the closed-chest group than in the open-chest group led to a longer, rather than a higher, exposure to noradrenaline before they were no longer able to generate sufficient pressure to maintain SAP above 50 mmHg and delay fWIT onset. Since catecholamine surges have been identified as a mechanism for inducing splenic red blood cell release in pigs (17), this longer catecholamine exposure might explain the significantly higher total hemoglobin level at fWIT in the closed-chest group than in the open-chest group. Furthermore, this mechanism of a longer duration between WLST and fWIT is consistent with the tendency for a greater increase in circulating free fatty acids in closed-chest pigs.

**Figure 8:**
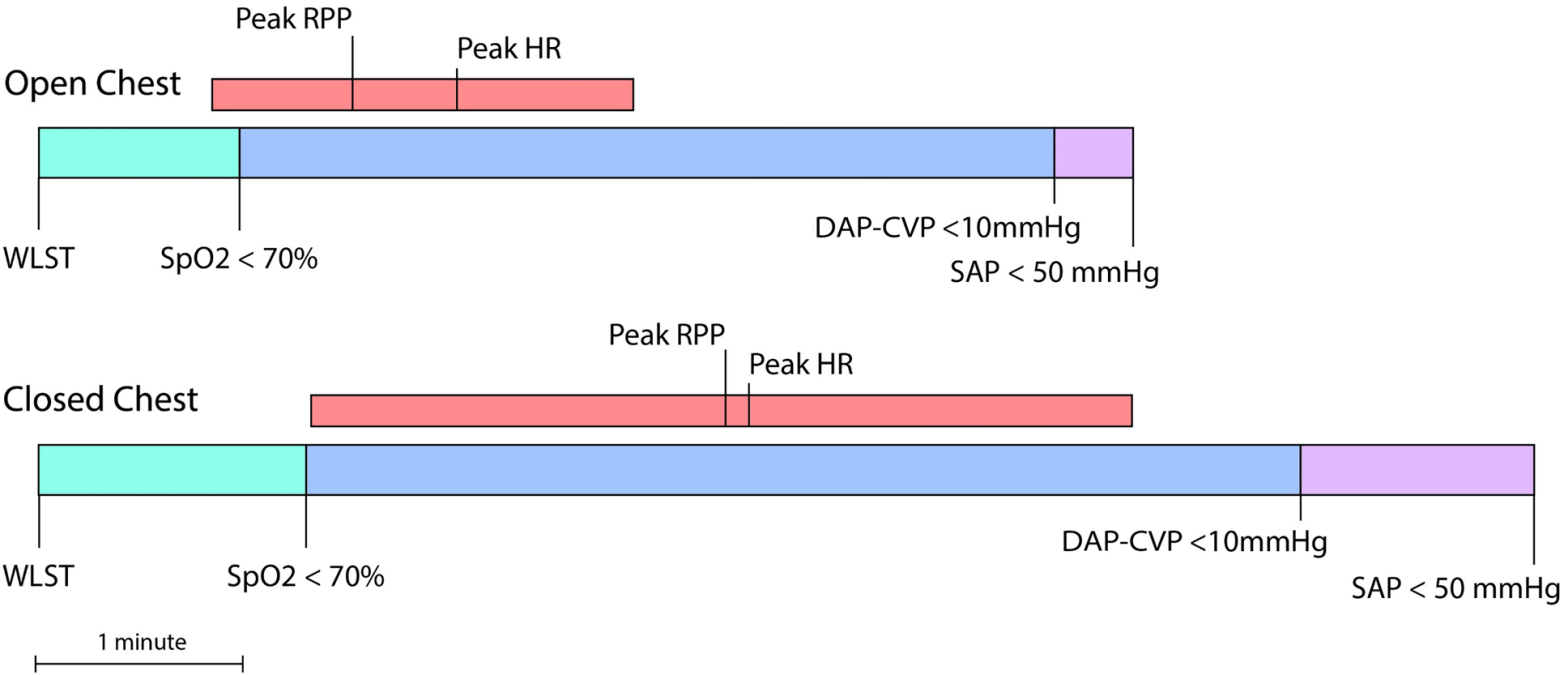
Progression from WLST to fWIT onset (SAP < 50 mmHg) in the open- vs. closed-chest models of DCD. DCD cardiac grafts are subjected to potentially damaging conditions associated with donor death prior to procurement, including hypoxia, hemodynamic instability, and warm ischemia. Importantly, the heart, with its high-energy demand, is particularly sensitive to warm ischemia, the duration of which is a critical factor in the decision of whether to proceed with the transplantation of a DCD graft. As such, the fWIT duration in the clinical setting is limited to 30 min. However, universal definitions for the start of fWIT are applied neither in clinical nor preclinical settings. We report that hearts under open-chest conditions undergo a significantly shorter period of hypoxia between 70% SpO_2_ and fWIT onset than those in the closed-chest group. Once the difference between DAP and CVP falls below 10 mmHg, the pressure gradient is insufficient to permit coronary perfusion. We also observed that the interval between DAP − CVP < 10 mmHg and fWIT onset tends to be longer in the closed-chest condition than in the open-chest condition. Thus, the hearts in an open-chest setting are less exposed to a shorter period of potentially damaging conditions than those in a closed-chest setting. These findings indicate that caution should be taken when interpreting results from open-chest preclinical models and translating these findings into clinical practice. RPP, rate-pressure product (HR × SAP).

The duration of warm ischemia is a crucial factor in deciding whether to proceed with transplantation of a DCD graft. Based on data from both clinical and preclinical reports (2, 18, 19), approximately 30 min of warm ischemia has been widely accepted in the clinic as the maximum tolerable time (14). However, exact start and end points for warm ischemia are not uniformly defined (7). This lack of consensus also applies to preclinical studies in DCD heart transplantation (8). Here, we defined the onset of fWIT as a drop in systolic blood pressure below 50 mmHg. However, it is clear that SpO_2_ levels substantially decrease prior to the onset of fWIT by this definition, and this supports the concept that the heart is already hypoxic at the point of fWIT onset (when SAP drops below 50 mmHg). Thus, hearts in the open-chest group undergo a significantly shorter period of hypoxia between 70% SpO_2_ and FWIT onset than in the closed-chest group. Furthermore, we provide evidence that coronary hemodynamics are compromised prior to fWIT onset. Zero-flow pressure in pigs has been reported as a difference of 6–9 mmHg between DAP and CVP during maximum vasodilation (20). We observed that the interval between a DAP − CVP < 10 mmHg and fWIT onset tended to be longer in the closed-chest group than in the open-chest group. Indeed, in the open-chest group, a DAP − CVP < 10 mmHg was reached approximately 30 s before fWIT onset; however, in the closed-chest group, the interval between DAP − CVP < 10 mmHg and fWIT onset was approximately twofold longer, as illustrated in Figure 8, a substantial time difference given the critical consequences of warm ischemia. As such, using a threshold of 50 mmHg SAP might underestimate fWIT duration, an argument against using 50 mmHg as a standalone criterion for fWIT start, in accordance with previous reports (11) and especially when a closed-chest model is used. Clinical transplant programs, such as that of St. Vincent's Hospital in Sydney (Australia), have reported using 90 mmHg as a threshold for the onset of fWIT (2). Although useful in humans, a systolic blood pressure of 90 mmHg in pigs is too high to distinguish from normal values (21), especially considering the animals are anesthetized. A possible solution to this might be the application of combined measures, for example, using systolic pressure in addition to SpO_2_. This approach has been implemented in the multi-center DCD Heart Trial (USA), which defines fWIT as SAP < 50 mmHg or peripheral saturation <70% (22). Regardless of the definition used for fWIT onset, differences remain between open- and closed-chest models with respect to progression from WLST to fWIT and the corresponding conditions to which hearts are exposed.

Significant changes in blood chemistry and circulating metabolites occurred throughout the experiment. For example, FFA levels increased after heparinization. This is likely a result of heparin-induced activation of lipoprotein lipase, which stimulates the release of free fatty acids into the circulation (23). Other than FFA, there were no significant changes in the assessed blood parameters between the baseline sample and the heparin sample, which indicates a stable pre-withdrawal phase and adequate anesthetic depth (24). FFA concentration remained high at the onset of fWIT, at which time the additional effect of elevated catecholamines would be expected (25). Circulating concentrations of glucose and lactate were also elevated at fWIT onset, albeit remaining within normal ranges. Circulating metabolites are of particular interest with regard to the *ex situ* phase of heart transplantation, given that donor blood is harvested for use during machine perfusion. High pre-ischemic FFA levels have been linked to poor recovery of the heart upon reperfusion in rodent experiments, where even brief exposure to circulating high fatty acids before ischemia led to a 50% decrease in hemodynamic recovery compared to hearts not exposed to high fat (26). Furthermore, increases in pre-ischemic lactate levels have been reported as detrimental to post-ischemic cardiac recovery in a rat model of DCD (27). The negative impact of these circulating metabolites renders them a target of interest for *ex situ* therapies. For example, in an ischemic environment, cardiac myocytes are subject to lipotoxicity, which stems from fatty acid uptake exceeding fatty acid oxidation (28), and the resulting mitochondrial dysfunction and cell death induction could possibly be prevented by stimulating fatty acid metabolism during reperfusion (24). In contrast to FFA, markers of myocardial cell death were not significantly increased at fWIT. Although these heart enzymes have a high sensitivity for myocardial damage in the context of acute myocardial infarction, their increase is usually seen within a much longer timeframe than what we report here (29).

This report provides initial evidence to support the concept that DCD donor organs are exposed to conditions that potentially differ in their severity according to open- or closed-chest conditions. However, additional studies are required to further characterize these changes; information on more specific cardiac damage, such as endothelial cell activation or injury or inflammatory responses, would be of value in assessing graft quality. Furthermore, previous research has suggested a potential higher tolerance for ischemia-reperfusion injury in females (30–34). However, how these differences translate to the context of the immediate post-WLST phase and progression to fWIT in DCD is not clear. Future experiments investigating perimortem physiology in DCD donors should consider exploring sex and gender differences, which might lead the way for more tailored recommendations regarding tolerable ischemic times and thresholds, and for the optimization of therapeutic strategies.

### Limitations

As a most notable difference from the clinical situation, the pigs did not suffer severe brain damage before WLST compared to a vast majority of human DCD donors. Thus, the effects of brain injury on the cardiovascular and respiratory systems and agonal breathing patterns could not be taken into consideration. Antemortem administration of heparin, as we performed, is not universally permitted in clinical practice (10). For the evaluation of oxygen saturation, the data from a pulse oximeter were used, which is less accurate under hypoxic conditions (35). Histopathological analysis of heart tissue was not considered for the comparison between groups, as histological samples were obtained after differing durations of ischemia; hearts in the open-chest group were not subjected to a warm ischemia period, while hearts in the closed-chest group underwent a period of warm ischemia. This study was conducted exclusively on male animals, precluding analysis of potential physiological sex differences during DCD and post-WLST.

## Conclusions

In this characterization of DCD pig models under both open- and closed-chest conditions, we demonstrate significant differences in perimortem physiology between these two models. Pigs undergoing sternotomy prior to WLST exhibited an accelerated decline in vital parameters and earlier onset of fWIT. Both groups followed the expected hyperdynamic response between WLST and fWIT; however, this response occurred earlier and tended to be less intense in the open-chest conditions than in closed-chest conditions. In addition, changes in blood chemistry also tended to be less severe under open-chest condition than in the closed-chest conditions. Taken together, our data support the concept that the conditions to which hearts are exposed in an open-chest model are potentially less damaging to the heart than those in a closed-chest model. As previous studies in porcine models of DCD have often been performed with sternotomies prior to WLST to allow for instrumentation (10, 11), it is important to interpret these results with care and to further validate findings in closed-chest models before translation into clinical practice.

## Data Availability

The raw data supporting the conclusions of this article will be made available by the authors without undue reservation.
